# The consistent burden in published estimates of delirium occurrence in medical inpatients over four decades: a systematic review and meta-analysis study

**DOI:** 10.1093/ageing/afaa040

**Published:** 2020-04-02

**Authors:** Kate Gibb, Anna Seeley, Terry Quinn, Najma Siddiqi, Susan Shenkin, Kenneth Rockwood, Daniel Davis

**Affiliations:** 1 MRC Unit for Lifelong Health and Ageing at UCL, Department of Population Science and Experimental Medicine, London, UK; 2 Acute Medical Unit, University College London Hospitals NHS Foundation Trust, UK; 3 Department of Medicine for the Elderly, University College London Hospitals NHS Foundation Trust, UK; 4 Institute of Cardiovascular and Medical Sciences, University of Glasgow, UK; 5 Department of Health Sciences, University of York, UK; 6 Department of Geriatric Medicine, University of Edinburgh, UK; 7 Department of Medicine, Dalhousie University, Halifax, Canada

**Keywords:** delirium, epidemiology, systematic review, meta-analysis, older people

## Abstract

**Introduction:**

Delirium is associated with a wide range of adverse patient safety outcomes, yet it remains consistently under-diagnosed. We undertook a systematic review of studies describing delirium in adult medical patients in secondary care. We investigated if changes in healthcare complexity were associated with trends in reported delirium over the last four decades.

**Methods:**

We used identical criteria to a previous systematic review, only including studies using internationally accepted diagnostic criteria for delirium (the Diagnostic and Statistical Manual of Mental Disorders and the International Statistical Classification of Diseases). Estimates were pooled across studies using random effects meta-analysis, and we estimated temporal changes using meta-regression. We investigated publication bias with funnel plots.

**Results:**

We identified 15 further studies to add to 18 studies from the original review. Overall delirium occurrence was 23% (95% CI 19–26%) (33 studies) though this varied according to diagnostic criteria used (highest in DSM-IV, lowest in DSM-5). There was no change from 1980 to 2019, nor was case-mix (average age of sample, proportion with dementia) different. Overall, risk of bias was moderate or low, though there was evidence of increasing publication bias over time.

**Discussion:**

The incidence and prevalence of delirium in hospitals appears to be stable, though publication bias may have masked true changes. Nonetheless, delirium remains a challenging and urgent priority for clinical diagnosis and care pathways.

## Key points

- The prevalence and incidence of delirium do not appear to have changed over the last four decades.
- Stable figures may result from better delirium care despite increased healthcare complexity.
- Publication bias increased over time.
- The entire delirium care pathway, from risk recognition, diagnosis, prevention and management, remains a key priority.

## Introduction

Delirium is characterised by the disturbance of consciousness and inattention triggered by an acute event (e.g. medical illness, surgery) [1]. It is substantially underdiagnosed in clinical practice, with a recent UK study demonstrating only 34% of older adults with delirium being recognised in routine clinical care [2]. This may partly be driven by its fluctuating nature and the diversity of clinical manifestations. It is associated with a wide range of adverse outcomes, particularly those relevant to patient safety. These include: mortality, falls, increased length of stay, and risk of institutionalisation [3,4]. In longitudinal studies, dementia is the biggest risk factor for delirium, and reciprocally, delirium is linked with worsening cognitive decline and incident dementia [5,6].

That delirium was a substantial burden among hospitalised older adults, which was established in a 2006 systematic review, describing delirium prevalence as ranging from 10 to 31% across 42 studies since 1980 (when delirium was first formally defined in DSM-III) [7]. Subsequently, a number of initiatives confirmed the need for better delirium prevention and management [8,9]. This increased focus on delirium coincided with gradual changes in the average patient age, background hospital prevalence of dementia and higher care complexity in patients admitted to hospital [10,11]. These underlying trends would be expected to lead to increases in delirium presentations, though this has never been directly investigated. Contemporary estimates of delirium epidemiology are needed, with implications for identifying training needs, clinical practice and public health policy [12]. In view of this, we set out to update the original systematic review in order to describe any change in the prevalence or incidence of delirium in the context of healthcare developments over the last four decades.

## Methods

### Eligibility criteria

We used identical criteria to the previous review [7], in line with PRISMA guidance [13]. As with the previous review, we considered prospective cohort and cross-sectional studies describing delirium in adults (aged 18 or older) who were acute, unscheduled admissions (including stroke and oncology patients) in any country and in any language. We did not include randomised controlled trials if we were unable to estimate cases in an unselected denominator. We excluded studies in terminally ill patients and those solely in patients referred to liaison psychiatry services. Studies in purely surgical cohorts, psychiatric units, emergency departments, coronary and intensive care units were excluded; studies in mixed populations were included if they separately reported information on internal medicine inpatients. Settings outside acute hospitals were excluded, for example post-acute care units, rehabilitation units, hospices and specialist palliative care units, and community hospitals. Reports on delirium specific to a clinical setting were excluded: e.g. delirium tremens, emergence delirium, post-electroconvulsive therapy and post-head injury. We only included peer-reviewed publications (i.e. we excluded abstracts and grey literature). Given this was an update of a previous systematic review, we did not devise a *de novo* protocol for PROSPERO.

### Outcome measures

We included studies which diagnosed delirium according to an internationally acceptable reference standard. Therefore, we considered diagnoses made by the Diagnostic and Statistical Manual of Mental Disorders (DSM) or the International Statistical Classification of Diseases (ICD), but not non-diagnostic screening instruments such as the Confusion Assessment Method [14]. To be included, ascertainment needed to have been performed by a person trained to apply the relevant reference standard (e.g. geriatrician, psychiatrist, nurse specialist and researcher); studies relying on routine clinical ascertainment were excluded. Studies where participants were pre-screened with a non-diagnostic tool prior to applying DSM or ICD to those screening positive for delirium were also excluded unless a sample of screen-negatives were also assessed.

Using an established operationalised reference standard is essential to investigate change over time, though different iterations of these classifications are inevitably also subject to temporal trends. Of the 42 cohorts included in the original review, we carried forward 15 studies that met this eligibility criterion. The 27 cohorts excluded at this stage included studies using unstandardised or non-diagnostic tools for which comparisons over time would be unreliable (Brief Psychiatric Rating Scale, Confusion Assessment Method, Memorial Delirium Assessment Scale, Delirium Rating Scale, Mini-Mental State Examination, Mental Status Questionnaire, Organic Brain Syndrome Scale and Short Portable Mental Status Questionnaire).

In describing the epidemiology of delirium in hospitals, prevalence conventionally refers to delirium ascertained on admission; incidence refers to delirium developing at some point over the inpatient admission. Where these have been difficult to distinguish—due to delirium fluctuations and/or different frequencies of observation—the more neutral term occurrence has usually been used. We considered studies which assessed the prevalence, incidence or occurrence of delirium.

### Search strategy

Updating the original review, we searched from 1 year prior to the previous end date (July 2004) to 31 May 2019. We searched the same electronic databases: Medline, EMBASE, PsycINFO, CINAHL Plus and the Cochrane Database of Systematic Reviews, using the following search terms; Delirium [Title] AND (epidemiology OR prevalence OR incidence OR occurrence) [Title/Abstract]. This replicated the original search strategy (provided through personal communication with the authors) except for the specification of the term ‘Delirium’. We chose not to include the various synonyms for delirium (e.g. acute confusional state, toxic psycosis) used in the previous search strategy because we were only interested in studies able to formally define delirium through a recognised reference standard. We confirmed the sensitivity of the search by ensuring that all studies from the previous review were captured.

### Data collection and study selection

Covidence (www.covidence.org, Veritas Health Innovation Ltd.) was used to manage the abstract and full text screening, assessment of risk of bias and data extraction. Titles and abstracts were independently reviewed by two reviewers (K.G. and A.S.) to determine the eligibility for inclusion. Conflicts were resolved by discussion and consensus. Data extraction for primary outcome and key variables was also performed by two reviewers (K.G., A.S. or D.D.) using a pro forma.

### Assessment of quality and biases

There is no consensus on the best tool for assessing risk of bias in descriptive epidemiology. The previous review used adapted criteria developed by the original authors [15]. We extended this previous approach by also accounting for items referred to in the Standards of Reporting of Neurological Disorders (STROND) criteria. [16,17] Ultimately, we considered five domains: (i) patient setting, e.g. general medical versus stroke patients; (ii) sample selection, e.g. randomised or convenience approach; (iii) sample criteria, e.g. exclusions based on capacity to consent or language; (iv) use of a defined reference standard and (v) expertise of assessor applying reference standard. In our assessment, we included articles from the original systematic review, so all findings reported here were considered with the same quality criteria. Each criterion was independently graded as low, medium or high risk of bias and we visualised this using the robvis package [18]. We described the certainty of our findings using an approach based on the GRADE framework, where we assessed risk of bias; consistency of results (based on heterogeneity); directness (applicability of included studies to research question); precision (based on confidence intervals of summary estimate) and publication bias (based on funnel plot). [19] To assess for temporal trends, we compared absolute values of publication bias estimates by decade.

### Statistical analyses

We extracted summary statistics for prevalence, incidence and occurrence, along with their standard errors. We anticipated methodological heterogeneity across cohorts, so accounted for this by calculating pooled estimates using DerSimonian–Laird random effects models. [20] Statistical heterogeneity was assessed with the I^2^ statistic. Meta-regression was used to estimate change over time and we used linear regression to examine if studies varied in average age or dementia prevalence in the samples, by year of publication. To assess publication bias, we plotted the estimated proportion of delirium occurrence against the standard error of that estimate, with Egger regression quantifying the degree of asymmetry. Stata 14.1 (StataCorp, Texas) was used for all analyses.

## Results

The search identified 4,137 citations of potential relevance. After removing duplicates, we screened 3,093 titles and abstracts and assessed 189 for full text review for eligibility (Supplementary data, Appendix 2). Full text screening excluded 171 studies; 50 were conference abstracts, 52 used methods other than DSM or ICD to diagnose delirium and 14 were studies of patient population not of interest, e.g. surgical, intensive care patients. All reasons for exclusion are detailed in Supplementary data, Appendix 2. We included 18 studies in this update, adding to 15 from the original review, to consider 33 studies altogether.

### Study characteristics

**Figure 1:**
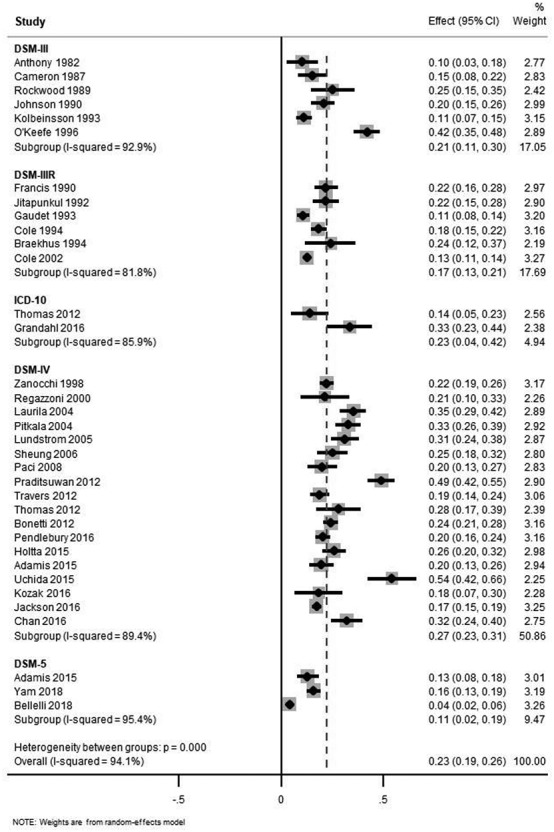
Meta-analysis of included studies (with studies from original review), stratified by diagnostic criteria and ordered by publication date. Note: Adamis (2015) and Thomas (2012) report prevalence by two diagnostic criteria in the sample but are weighted as separate studies.

All studies were carried out in acute medical or geriatric medicine units, and all were prospective cohort studies, except one cross-sectional study (Table 1). Most were conducted in Western European populations, though single studies from China, Turkey and Thailand were included. Studies ranged in size from *n* = 60 to 1,327 and varied in age (range of average sample age from 66 to 87 years) and prevalence of co-morbid dementia (range 8–100%). Delirium was diagnosed using DSM-IV or DSM-5 in 16 studies, and two used ICD-10, adding to the six using DSM-III, six using DSM-III-R and three using DSM-IV from the original review. Some studies reported estimates based on more than one criterion, therefore 35 occurrence estimates are included in Figure 1. These direct measures of delirium occurrence in a range of studies led to GRADE assessments of ‘not serious’ for indirectness and imprecision (Supplementary data, Appendix 1).

**Table 1:** Characteristics of included updated studies

Study	Country	Sample	Exclusion criteria	*N*	Mean age (years)	Dementia prevalence	Reference standard
Adamis, 2015	Ireland	≥70 years; all acute medical admissions	Hospitalised for >48 hours; readmitted to unit; studied on previous admission; severe aphasia; intubated; sensory problems; non-English speaking	200	81.1	63%	DSM-IV, DSM-5
Bellelli, 2018	Italy	≥70 years, consecutive admissions (multiple hospitals)	No proxy available for consent	588	80.9	12%	DSM-5
Bonetti, 2012	Italy	>64 years; admissions to geriatric units	Nil	578	82	NR	DSM-IV
Chan, 2016	China	≥18 years; admissions to the respiratory wards for acute respiratory failure with non-invasive positive pressure ventilation	Persistent coma; those who lacked mental capacity to provide consent and guardian not available; unavailable in first 48 hours of admission (died or discharged)	153	74.2	7.8%	DSM-IV
Grandahl, 2016	Denmark	≥18 years; admission to oncologic ward; histologically verified cancer diagnosis	Non-Danish speaking patients; readmitted to unit; studied on a previous admission	81	68.5	NR	ICD-10
Holtta, 2015	Finland	≥70 years; admissions to acute geriatrics wards	Coma	255	86.6	100%	DSM-IV
Jackson, 2016	UK	≥70 years; admissions to acute medicine	Unable to communicate because of severe sensory impairment; unable to speak English; at risk of imminent death	1327	84.4	36%	DSM-IV
Kozak, 2016	Turkey	≥18 years; clinical presentation of acute ischaemic stroke	Admission to hospital after first 24 hours; a diagnosis of TIA, cerebral haemorrhage; reduced GCS, severe aphasia or dysphasia; history of brain tumour, myocardial infarction, infection, autoimmune and immunosuppression, recent trauma or surgery; renal dysfunction and symptomatic peripheral arterial disease; GI or rheumatic inflammatory disease, metabolic syndrome; recent antidepressant use	60	66.2	NR	DSM-IV
Laurila, 2004	Finland	≥70 years	Coma	219	≥85 = 59%	40%	DSM-IV
Paci, 2008	Italy	Stroke; admissions to the stroke unit during the first 5 days of hospitalisation	Nil	150	67.5	NR	DSM-IV
Pendlebury, 2015	UK	Admissions to acute medical unit	Nil	503	72 (median)	10%	DSM-IV
Pitkala, 2004	Finland	≥70 years	Coma	230	≥85 = 62%	61%	DSM-IV
Praditsuwan, 2012	Thailand	≥70 years; admissions to general medical wards	Endotracheal intubation at admission; aphasia; uncooperative; coma	225	78	42%	DSM-IV
Sheung, 2006	Australia	≥65 years; admissions with acute stroke	TIAs; subarachnoid haemorrhage; history of severe head trauma or neurosurgery before stroke; stroke due to tumour or cerebral venous sinus thrombosis	156	79.2	7.7%	DSM-IV
Thomas, 2012	Germany	≥80 years; admissions to geriatric unit	Global aphasia; terminal condition	79	84.1	75%	DSM-IV, ICD-10
Travers, 2012	Australia	≥70 years; admissions to general medical and surgical wards; expected hospitalisation >48 hours	Transferred to a study ward from another hospital or ward and admitted for >48 hours previously; immunocompromised; imminent death	294	80.4	26%	DSM-IV
Uchida, 2015	Japan	≥65 years; incurable lung or GI cancer; planned admission of ≥2 weeks; performance status of 2 or worse	Physically too ill to complete the survey; non-Japanese speaking	61	72	NR	DSM-IV
Yam, 2018	China	≥65 years; admissions to general medical wards	Direct admissions to the intensive care unit, coronary care unit and acute stroke unit; coma, persistent vegetative state; severe aphasia; clinically unstable; deemed too unwell	575	80.8	NR	DSM-5

NR: not reported. Note some sample overlap is possible between Pitkala (2004) and Laurila (2004).

**Figure 2:**
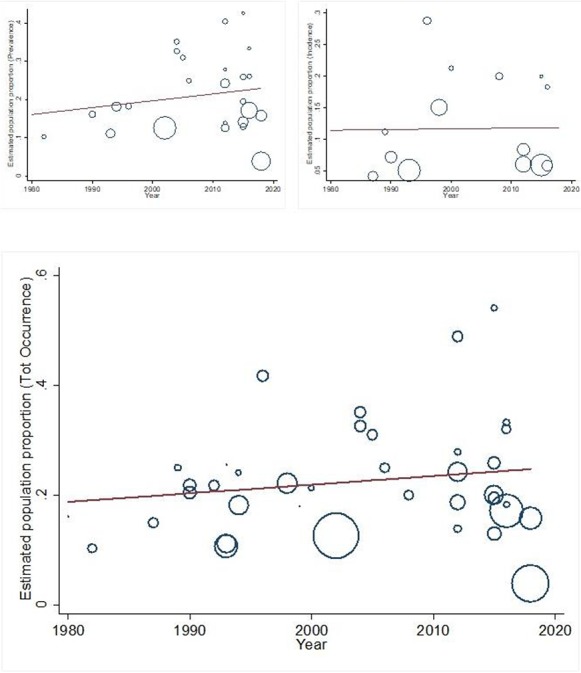
(a–c) Temporal trends in delirium prevalence (top left), incidence (top right) and occurrence (bottom).

### Study quality

Sources of risk of bias were assessed in all studies (including from the original review) according to the domains detailed in Supplementary data, Appendix 3. Studies scored ‘low risk’ or ‘some concerns’ in all domains, with 27 of 33 studies considered to be low risk overall (GRADE assessment low, Supplementary data, Appendix 1). Most studies were rated ‘some concerns’ for source population because the sample was from a single centre (Domain 2, Supplementary data, Appendix 3). Other studies had potential sources of bias through excluding people with severe aphasia, inability to communicate due to severe sensory problems, those lacking capacity to consent (or no provisions for proxy consent), terminally ill or in coma (Domain 3, six studies).

### Delirium prevalence, incidence and occurrence

Pooled prevalence was estimated as 15% (95% CI 14–16%, 25 studies). Cumulative incidence of new delirium was 9% (95% CI 7–10%, 14 studies) over the observed period, which was up to 2 weeks in duration. Figure 1 shows estimates of the total delirium occurrence of 23% (95% CI 19–26%), stratified by reference standard. There was a wide range in estimates, from 4 to 54%. Differences in occurrence estimates were evident according to diagnostic criteria, with DSM-IV and DSM-5 showing higher and lower estimates, respectively. These different criteria over time led us to assign a GRADE inconsistency rating of ‘serious’ (Supplementary data, Appendix 1).

Figure 2a–c indicates the prevalence, incidence and occurrence over time (1980–2019). Meta-regression models did not demonstrate any statistically significant temporal changes (prevalence: increasing by 0.2%/year, 95% CI −0.2 to 0.6%/year, *P* = 0.38; incidence: −0.1%/year, 95% CI −0.4 to 0.4%/year, *P* = 0.95; occurrence: 0.2%/year, 95% CI −0.2 to 0.5%/year, *P* = 0.35).

**Figure 3:**
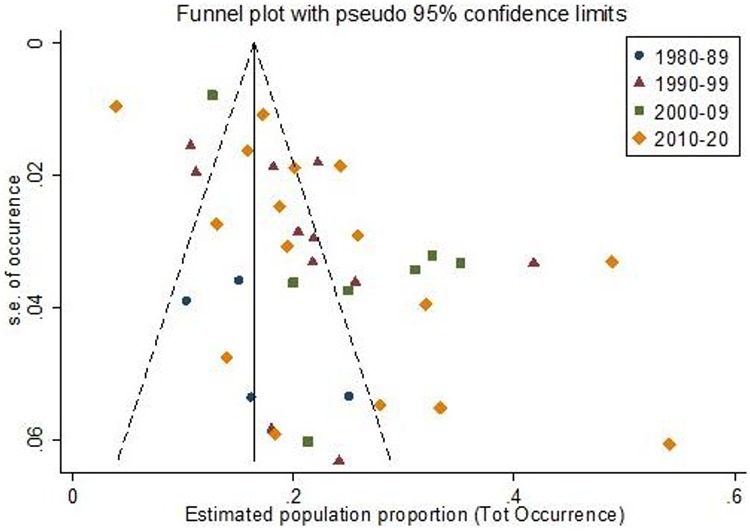
Funnel plot showing the occurrence of delirium in relation to standard error of the estimate, by decade.

Over time, there were no differences in the average age of the samples in included studies (mean age across studies 80.0 years, change over time −0.28/year, 95% CI −0.79 to 0.24, *P* = 0.28). Where studies indicated the prevalence of comorbid dementia in the sample (*n* = 19), these also did not show any changes over the study period (mean prevalence of dementia 40%, change over time 0.11%/year, 95% CI −0.02 to 0.23%, *P* = 0.10).

### Publication bias

Publication bias was suggested from asymmetry in forest plots (Egger coefficient 5.10, *P* < 0.01, Figure 3). However, this was not shown in the earlier studies and more funnel plot asymmetry was apparent from 2000 onwards (1980–89 coefficient 4.24, *P* = 0.32; 1990–99 coefficient 4.22, *P* = 0.09; 2000–09 coefficient 5.08, *P* = 0.02; 2010–19 coefficient 5.99, *P* = 0.01).

## Discussion

According to this systematic review and meta-analysis, the published prevalence and incidence of delirium in acute medical adult inpatients have remained broadly stable at about one in four older patients. We quantified this from studies using consistent methods in comparable populations. There were no major differences in aspects of the case mix described in the studies (average age, dementia prevalence) across time, though other relevant factors such as frailty were not reported in sufficient detail to be addressed here. There was evidence for increasing publication bias, suggesting that estimates supporting a higher apparent burden of delirium are more likely to be published; these samples may not be representative of clinical patients in routine care. Taken together, delirium remains a substantial problem in acute hospitals, though quantifying this in relation to increased healthcare complexity alongside increased prioritisation of delirium in clinical practice is not straightforward (GRADE recommendation ‘moderate’, Supplementary data, Appendix 1).

Several limitations to our findings require further comment. To be consistent with the original review, we only considered studies on acute medical and geriatric medicine inpatients. This limits generalisability to other settings. We could not account for illness severity nor were direct measures of frailty available. While it is clear that most delirium risk is conferred by age and baseline dementia status, it is likely that more nuanced measures may have captured changes in case mix more accurately. We expected to see variation in case mix across time; at least for average age and underlying dementia prevalence, this did not appear to change. Methods to ascertain dementia prevalence in hospitals were themselves heterogeneous across studies, where reported, though in the main they were defined by researchers rather than relying on routine detection. Finally, different iterations of the DSM criteria have different degrees of inclusivity for defining delirium [21]. It is worth noting that as studies using DSM-5 become more common, future case ascertainment will depend on strict versus relaxed interpretations of the criteria [22].

To an extent, publication bias may account for some of these trends. The funnel plot asymmetry demonstrates that smaller studies are more likely to have higher estimates of delirium occurrence than would be expected by chance. This could be due to the lack of drive to publication from anywhere in the research process, including studies finding low delirium prevalence to submitting for publication at all due to a perception that such findings will be of less interest. Because the asymmetry increases with each decade, it is possible that researchers are only submitting (and journals publishing) results consistent with this perception that delirium is common. If as a consequence, these are less representative of clinical patient populations, then prevalence and incidence of delirium may be being overestimated in our included studies. Other aspects to the risk of bias assessments indicated that our findings were not subject to much variation due to the training of the diagnostic rater, or particularly limited by selection bias because of inappropriate exclusions.

To highlight the overall clinical implications, no net change in the reported epidemiology confirms delirium as a major healthcare concern. In particular, rates of incident delirium remain high, suggesting that front-door preventative measures have not made substantial impact in public health terms. However, there is also the possibility that diverging trends underlie our findings. On the one hand, increasing complexity of healthcare and frailty among acute admissions may lead to more delirium. In contrast, delirium has attracted much more prominence in recent years with increased emphasis on multicomponent prevention [23], representation in clinical care pathways and guidelines [12] and recognition of its potential role in dementia prevention [24]. There is some suggestion that clinical pathways for delirium may have been effective in the context of acute stroke services [25]. However, if the publication bias leads to inflated estimates of delirium occurrence in more general settings, then the effectiveness of delirium prevention initiatives may be being masked.

The estimates presented in this review are based on research-grade ascertainment of delirium, yet there is a clear need to implement delirium detection in routine care while maintaining accuracy even when used at scale. For example, even with nearly 100% completion rates, the Confusion Assessment Method—a common delirium screening tool—performed twice daily was positive in only 2% of patients, far lower than the 17% rate found when delirium was measured by psychiatric assessment in the same clinical unit [26]. By contrast, in UK hip fracture patients, the 4AT delirium detection tool [27] had variable rates of post-operative completion (95% in England, 38% achieved in Wales and 42% in Northern Ireland), but around 25% of tests were positive, which is closer to the findings reported here [28]. Fundamentally, it remains the case for all delirium research that ascertainment procedures should be explicitly reported, specifically including the details of cognitive tests, thresholds for defining deficits and adjudication methods for borderline cases [29,30]. Nonetheless, it is clear that the extent of delirium remains considerable. There can be no complacency around prioritising the entire delirium care pathway, from risk recognition, diagnosis, prevention and management.

In this updated systematic review and meta-analysis, we found that the epidemiology of delirium among hospitalised patients has not changed substantially between 1980 and 2019. At least in estimates from the published literature, case mix also appears not to have changed much. With this burden of delirium in hospitals, contemporary priorities around disseminating delirium knowledge, increasing the proportion diagnosed and implementing care pathways remain as challenging yet urgent as ever.

## Supplementary Material

aa-19-0994-File002_afaa040
