# Supplementary material for: The consistent burden in published estimates of delirium occurrence in medical inpatients over four decades: a systematic review and meta-analysis study
Source: Age Ageing. 2020 Apr 2;49(3):352–60. doi: 10.1093/ageing/afaa040 (PMC7187871; doi:10.1093/ageing/afaa040)
Supplement: aa-19-0994-File002_afaa040 [file aa-19-0994-file002_afaa040.docx]

**The consistent burden in published estimates of delirium occurrence in medical inpatients over four decades: a systematic review and meta-analysis study**

**SUPPLEMENTARY MATERIAL**

**Appendix 1.** Grading of Recommendations, Assessment, Development and Evaluation (GRADE) assessment.

| Occurrence (incidence and prevalence) of delirium in general medical inpatients | | | | | | | |
| --- | --- | --- | --- | --- | --- | --- | --- |
| No. studies  Design | Risk of bias | Inconsistency | Indirectness | Imprecision | Publication bias | Effect  (95% CI) | Quality of evidence |
| 38 studies  Observational | Low | Serious | Not serious | Not serious | Serious | 23%  (95% CI 19%-26%) | Moderate |

**Appendix 2**: PRISMA flowchart of search results and study retrieval


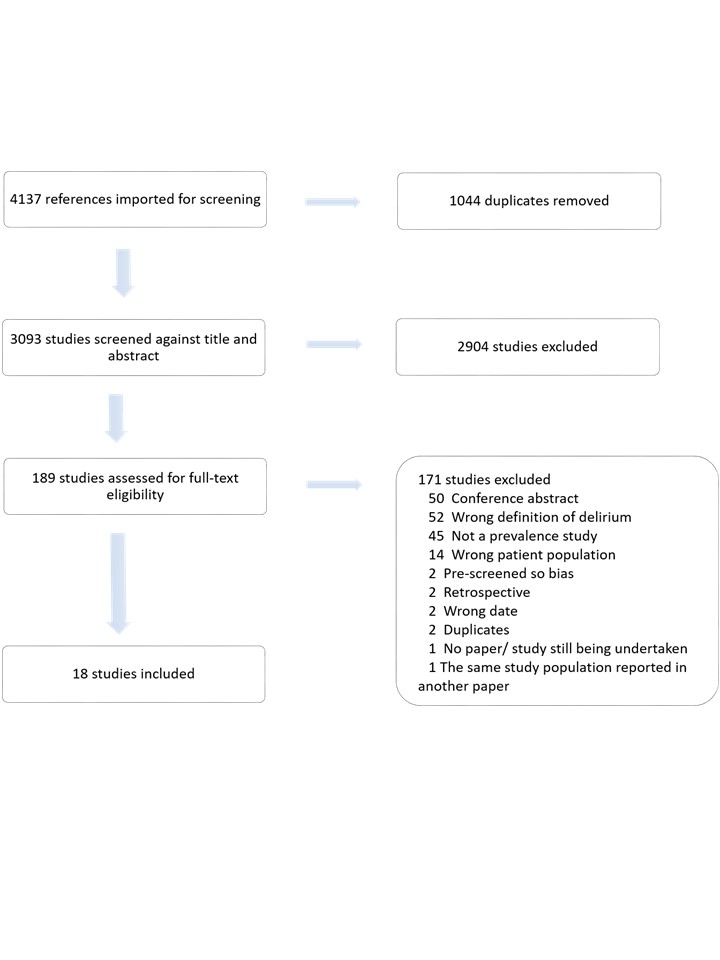


**Appendix 3.** Risk of bias assessments

**
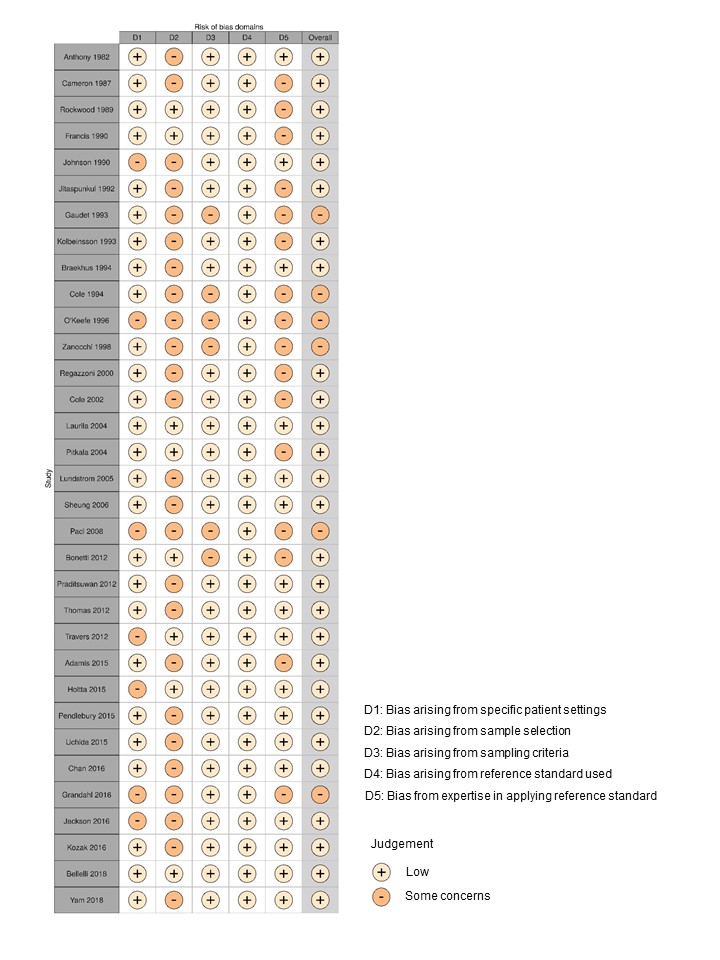
**

**
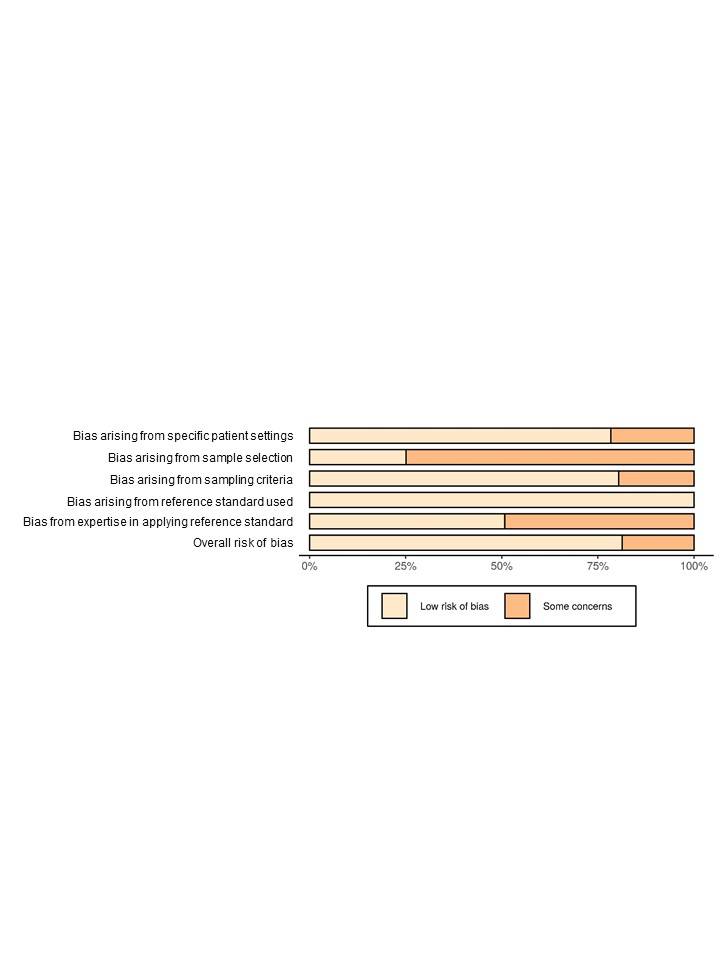
**

**Supplementary References**

1. Anthony JC, LeResche L, Niaz U, von Korff MR, Folstein MF. Limits of the 'Mini-Mental State' as a screening test for dementia and delirium among hospital patients. Psychol Med. 1982 May;12(2):397-408.

2. Cameron DJ, Thomas RI, Mulvihill M, Bronheim H. Delirium: a test of the Diagnostic and Statistical Manual III criteria on medical inpatients. J Am Geriatr Soc. 1987 Nov;35(11):1007-10.

3. Rockwood K. Acute confusion in elderly medical patients. J Am Geriatr Soc. 1989 Feb;37(2):150-4.

4. Johnson JC, Gottlieb GL, Sullivan E, Wanich C, Kinosian B, Forciea MA, et al. Using DSM-III criteria to diagnose delirium in elderly general medical patients. J Gerontol. [Research Support, Non-U.S. Gov't]. 1990 May;45(3):M113-9.

5. Kolbeinsson H, Jonsson A. Delirium and dementia in acute medical admissions of elderly patients in Iceland. Acta Psychiatr Scand. 1993 Feb;87(2):123-7.

6. O'Keeffe ST, Lavan JN. Predicting delirium in elderly patients: development and validation of a risk-stratification model. Age Ageing. 1996 Jul;25(4):317-21.

7. Francis J, Martin D, Kapoor WN. A prospective study of delirium in hospitalized elderly. JAMA : the journal of the American Medical Association. 1990 Feb 23;263(8):1097-101.

8. Jitapunkul S, Pillay I, Ebrahim S. Delirium in newly admitted elderly patients: a prospective study. Q J Med. 1992 Apr;83(300):307-14.

9. Cole MG, Primeau FJ, Bailey RF, Bonnycastle MJ, Masciarelli F, Engelsmann F, et al. Systematic intervention for elderly inpatients with delirium: a randomized trial. CMAJ. 1994 Oct 1;151(7):965-70.

10. Braekhus A, Engedal K. [Delirium (acute confusion) among elderly patients after admission to a medical department]. Tidsskr Nor Laegeforen. 1994 Sep 20;114(22):2613-5.

11. Cole MG, McCusker J, Dendukuri N, Han L. Symptoms of delirium among elderly medical inpatients with or without dementia. J Neuropsychiatry Clin Neurosci. 2002 Spring;14(2):167-75.

12. Zanocchi M, Vallero F, Norelli L, Zaccagna B, Spada S, Fabris F. [Acute confusion in the geriatric patient]. Recenti Prog Med. 1998 1998/05//;89(5):229-34.

13. Regazzoni CJ, Aduriz M, Recondo M. [Acute confusion syndrome in the hospitalized elderly]. Medicina (B Aires). 2000;60(3):335-8.

14. Gaudet M, Pfitzenmeyer, P., Tavernier-Vidal, B., Lechenet, M. [Delirium in acute care geriatric hospital.]. Psychologie Medicale. 1993;25(7).

15. Lundstrom M, Edlund A, Karlsson S, Brannstrom B, Bucht G, Gustafson Y. A multifactorial intervention program reduces the duration of delirium, length of hospitalization, and mortality in delirious patients. J Am Geriatr Soc. 2005 Apr;53(4):622-8.

16. Adamis D, Rooney S, Meagher D, Mulligan O, McCarthy G. A comparison of delirium diagnosis in elderly medical inpatients using the CAM, DRS-R98, DSM-IV and DSM-5 criteria. International Psychogeriatrics. 2015 04 Jun;27(6):883-9.

17. Bonetti F, Magon S, Gasperini B, Zampi E, Cerenzia A, Vergani V, et al. Risk factors associated to delirium in hospitalized elderly patients. Giornale di Gerontologia. 2012 June;60(3):142-8.

18. Chan KY, Cheng LSL, Mak IWC, Ng SW, Yiu MGC, Chu CM. Delirium is a Strong Predictor of Mortality in Patients Receiving Non-invasive Positive Pressure Ventilation. Lung. 2017 01 Feb;195(1):115-25.

19. Grandahl MG, Nielsen SE, Koerner EA, Schultz HH, Arnfred SM. Prevalence of delirium among patients at a cancer ward: Clinical risk factors and prediction by bedside cognitive tests. Nordic Journal of Psychiatry. 2016 17 Aug;70(6):413-7.

20. Holtta EH, Laakkonen ML, Laurila JV, Strandberg TE, Tilvis RS, Pitkala KH. Psychotic symptoms of dementia, their relationship with delirium and prognostic value. European Geriatric Medicine. 2015 01 Jun;6(3):257-61.

21. Jackson TA, MacLullich AMJ, Gladman JRF, Lord JM, Sheehan B. Undiagnosed long-term cognitive impairment in acutely hospitalised older medical patients with delirium: A prospective cohort study. Age and Ageing. 2016;45(4):493-9.

22. Kozak HH, Uguz F, Kilinc I, Uca AU, Serhat Tokgoz O, Akpinar Z, et al. Delirium in patients with acute ischemic stroke admitted to the non-intensive stroke unit: Incidence and association between clinical features and inflammatory markers. Neurologia i Neurochirurgia Polska. 2017 01 Jan;51(1):38-44.

23. Paci C, Gobbato R, Carboni T, Sanguigni S, Santone A, Coccia G, et al. Quetiapine responsive delirium in acute stroke. Rivista di Psichiatria. 2008 Mar-Apr;43(2):101-3.

24. Pendlebury ST, Lovett NG, Smith SC, Dutta N, Bendon C, Lloyd-Lavery A, et al. Observational, longitudinal study of delirium in consecutive unselected acute medical admissions: Age-specific rates and associated factors, mortality and re-admission. BMJ Open. 2015;5 (11) (no pagination)(e007808).

25. Praditsuwan R, Limmathuroskul D, Assanasen J, Pakdeewongse S, Eiamjinnasuwat W, Sirisuwat A, et al. Prevalence and incidence of delirium in Thai older patients: a study at general medical wards in Siriraj Hospital. Journal of the Medical Association of Thailand = Chotmaihet thangphaet. 2012 Feb;95 Suppl 2:S245-50.

26. Sheng AZ, Shen Q, Cordato D, Zhang YY, Chan DKY. Delirium within three days of stroke in a cohort of elderly patients. Journal of the American Geriatrics Society. 2006;54(8):1192-8.

27. Thomas C, Kreisel SH, Oster P, Driessen M, Arolt V, Inouye SK. Diagnosing delirium in older hospitalized adults with dementia: Adapting the confusion assessment method to International Classification of Diseases, Tenth Revision, diagnostic criteria. Journal of the American Geriatrics Society. 2012 Aug;60(8):1471-7.

28. Travers C, Byrne G, Pachana N, Klein K, Gray L. Prospective observational study of dementia and delirium in the acute hospital setting. Internal Medicine Journal. 2013 March;43(3):262-9.

29. Uchida M, Okuyama T, Ito Y, Nakaguchi T, Miyazaki M, Sakamoto M, et al. Prevalence, course and factors associated with delirium in elderly patients with advanced cancer: A longitudinal observational study. Japanese Journal of Clinical Oncology. 2015 October;45(10):934-40.

30. Yam KK, Shea YF, Chan TC, Chiu KC, Luk JKH, Chu LW, et al. Prevalence and risk factors of delirium and subsyndromal delirium in Chinese older adults. Geriatrics and Gerontology International. [In Press]. 2018.
